# Testimony of Medical Experts*Concluded from May Number.

**Published:** 1880-06

**Authors:** Charles Beckwith

**Affiliations:** Judge of the Superior Court of Buffalo, N. Y.


THE BUFFALO

MEDICAL AND SURGICAL JOURNAL.

Vol. XIX.—JUNE, 1880.—No. 11.

### Original Communications.

### TESTIMONY OF MEDICAL EXPERTS.*

BY HON. CHARLES BECKWITH,

Judge of the Superior Court of Buffalo, N. Y.

There is another question which has excited some interest recently, that is, the compensation to which medical and other scientific experts are entitled upon being required to testify in court. It is claimed by some that a physician or surgeon can not be required to testify to a matter that involves the employment of his professional knowledge or skill without recompense, or the promise of recompense, as for a professional service or opinion. Dr. Ordroneaux, in his work on the Jurisprudence of Medicine, affirms this proposition. Curiously enough the question seems not to have been much before the courts, either in this country or in England. That would indicate that the right to such compensation had been generally conceded or allowed; or, on the other hand, that the right to it had not been thought of, nor asserted, nor such compensation allowed. I think the annals of the legal and medical professions, as well as experience, tend to show that the allowance of such a compensation had never

* Concluded from May Number.

in fact become a practice. If I am correct in the view I have taken as to the nature of expert testimony, and its quality and rank as evidence, and its use in legal investigations, no claim to extra compensation by the witness could be sustained on account of any superiority in species of the testimony he gives over other testimony in the case, or on account of its greater importance to the courts in the administration of justice. We must look, then, for the reasons that substantiate such a claim, to the office of the expert, his situation in society, or his relations to the duty or act of giving his opinions in evidence in courts, as required by law. This question has not occupied the attention of the courts very much in England, owing probably to the existence of a statute passed in the reign of Queen Elizabeth, requiring the payment to a witness, when subpœnaed, of his reasonable charges, according to his "countenance and calling." In this State the law prescribes an invariable fee for all witnesses, high or low, rich or poor, which is fifty cents for each day's attendance; and if the witness reside more than three miles from the place of trial, four cents a mile travel-fees going and returning—thus placing, in this respect, all citizens of the State upon a level of equality. The people of this State have adopted a policy which is plainly visible in the statutes which they, in their majesty, have prescribed. They have empowered every court of record, in the language of the statute, "to issue process of subpœna, requiring the attendance of any witness residing, or being in any part of the State, to testify in any matter or cause pending in such court." Again the statute provides, "Every court of record shall have power to punish by fine and imprisonment or either, all persons summoned as witnesses, for refusing or neglecting to obey such summons, or to attend, or be sworn or answer as such witnesses." There are numerous other provisions for compelling the attendance of witnesses, and for punishing those who intercept or restrain them in going to court, and also making it unlawful even to arrest them, when under subpœna, on civil process.

Thus it would appear that under the policy of this State all persons indiscriminately are bound, when summoned by lawful mandate, to appear and testify in the courts. In the case of the People vs. Montgomery (13 Abb. Rep. (N. S.) 207) tried in the County of Monroe, in 1870, where a contract was entered into by one party to the action with a medical expert for a compensation of $500, the question arose as to whether the validity of the verdict was affected. It was held that it was not. The Court said: "The District Attorney, it is true, might have required the attendance of Dr. Hammond on subpœna; but that would not have sufficed to qualify him to testify as an expert, with clearness and certainty, upon the question involved." * * "He could not have been required, under process of subpœna, to examine the case and to have used his skill and knowledge to enable him to give an opinion upon any points of the case."

Under the authority of this decision it seems that if the law should be held to be, that a medical expert is bound to obey a subpœna, still, he has it in his individual power, if a party wants his opinion, to coerce compensation as for professional services, by refusing to examine the case before going to court, and by refusing to listen to and consider the testimony given on the trial. And it may be conceded that the court can not compel the witness to spend any time, or perform any examination or service in order to prepare himself to give testimony upon the facts, or to give an opinion. It is, however, contended in some quarters that a medical expert can not be compelled upon service of a subpœna, and tender of the ordinary witness' fee, to give his opinion or testimony, even in those cases, and upon those questions where no special preparation is necessary.

The question was up recently in the State of Indiana, in the case of Buchman vs. The State. Dr. Buchman appeared in court on the subpœna of the prisoner, and put on the stand as a witness. He testified that he was a physician and had practiced several years. A question was then propounded to him which he refused to answer, saying that the answer would depend upon his professional knowledge of the subject, and he would not give it without being paid. The trial court held that he was bound to answer the question without extra compensation, and the witness persisting in his refusal to answer, the court committed him for contempt. The Doctor took an appeal from the order committing him for contempt and carried the question to the highest court of review in that State. The Supreme Court held that his committment was error. The argument of the court was that physicians and surgeons, whose opinions are valuable to them as a source of income and livelihood can not be compelled to give their opinions in a court of justice upon professional questions without compensation as for professional services; that in testifying as an expert by giving his opinion, the physician is performing in reality a professional Service. The decision goes upon the ground that the professional knowledge and skill of a medical expert is his private property, and cannot be taken from him without compensation, and that giving his opinions on trials at law is the performance of a professional service.

It is the principle embodied in all the state constitutions that you shall not take a man's private property without compensation.

But how is it that a physician's property is taken from him when he gives his opinion as an expert in a court of justice?

Suppose his knowledge and skill, in a figurative sense, are his property and his capital stock in business, are they taken from him when he testifies? It seems to me one might as well say that a man parts with the grace and sentiment of charity by giving alms. What does he lose that is his own when he testifies? Does he not retain in possession the same knowledge and skill? If he does, then of what property has he been deprived? "By giving ye shall receive." It is the compensation of nature that every effort one puts forth, especially under the stimulus of important occasions, makes more clear and perfect his hold upon his mental treasures. And what ownership has the expert in those matters to which he testifies—those relations existing between special cases and general states; those sequences of cause and effect in physical facts; those logical truths of the agreement or disagreement of special phenomena with the supposed and accredited order of nature?

The Supreme Court of Indiana, certainly a court entitled to great respect, uses the further argument that the more eminent a physician or surgeon, the more frequently will he be called upon to go into all parts of the State, to render his services, without other compensation than the ordinary witness fees. In exceptional cases a hardship of this kind might occur, but I fail to see its force as an argument that professional compensation can be claimed. Men in all ranks feel the hardship of those compulsory sacrifices they make in the public interests. Under the operation of our statutes, Vanderbilt could to-day be summoned away from his imperial railway enterprises, to attend an ordinary trial in Buffalo, and along with him the poor artisan or laborer whose family depend for sustenance on his daily earnings.

The same question of the right of the surgeon to refuse to give his professional opinion until paid therefor, arose also not long since, in the case *ex parte Dement*, reported in the 53d volume of the Alabama reports. It was a trial of an indictment for murder, and Dr. Dement having been put on the stand as a witness, refused to answer a question that called for his opinion respecting the wounds found on the body of the murdered man, on the ground that he had not been compensated. The judge imposed a fine for contempt. Afterwards the doctor brought a proceeding in court to vacate the order fixing the fine, claiming that it had been illegally imposed, and the matter was carried to the court of last resort for review. The case was instituted, it is said, in the interests of the medical fraternity of that State. That court, after careful consideration, came to a conclusion directly opposite to that of the Supreme Court of Indiana, and held that the fine was lawfully imposed.

*Maning, J.*, delivering the opinion of the court, says: "The same principle which justifies the bringing of the mechanic from his workshop, the merchant from his store-house, the broker from 'change, or the lawyer from his engagements, to testify in regard to some matter which he has learned in the exercise of his art or profession, authorizes the summoning of a physician or surgeon, or skilled apothecary, to testify of a like matter, when relevant to a cause pending for determination in a judicial tribunal. And if in a prosecution for murder it was proved that his supposed victim had, a short time before his death, drank something which he had received from the accused, and a chemist had analyzed the liquid, and testified what substances it contained, and a physician was summoned to prove what effect they would have when taken into the stomach of a living man, and what would be the symptoms of such effect, no court would be excusable in exonerating the physician from giving such evidence, solely on the ground that it would be a professional opinion for which he had not been paid, or received a promise of payment. In so testifying he would not be practicing the healing art; he would, like the merchant, or the lawyer, or the mechanic, before referred to, be deposing only to those things which he had learned in the course of his occupation or profession, or of the preparation for it, and the disclosure of which to the court would conduce to a correct understanding of the cause before it. His testimony would concern the administration of justice, and of him as of the other witnesses, it could be justly claimed by the public as a tax paid by him to that system of laws which protects his rights as well as others."

As is apparent, the decision of the Alabama court meets my approval rather than that of the Supreme court of Indiana. While I am of the opinion that the medical expert in court has not now, in law, a right to professional compensation, still there may exist an equitable claim here that deserves legislative remedy, perhaps a return to the wisdom of Elizabeth, grading the compensation of witnesses according to their "countenance and calling."

Certainly the practice of allowing parties to contract with experts upon the consideration of round sums of money to prepare themselves and come into court to testify, to swear away the rights of their fellow-men, is a strange incongruity in that system which provides so many barriers against the influence upon the scales of justice of all forms of interest, prejudice, bias or passion. Since the trial of Dr. Schoeppe, in Pennsylvania, Mrs. Wharton in Maryland, and other equally notorious cases, one may well feel apprehensive about adding the thirst for gold to the ordinary and natural zeal of the professional witness. The inquiry suggests itself whether in important cases where the testimony of experts is necessary, the court should not have and exercise the power of appointing a commission of skilled persons to examine the facts and testify to the results and their opinion as *quasi* officers of the court, reserving always to the party affected adversely the right to require an exposure of the facts and the grounds of the opinions.

